# UTILITY OF THE CARE TRANSITION MEASURE (CTM-15) AND A MEASURE OF CONTINUITY OF CARE IN LIVE DISCHARGE

**DOI:** 10.1093/geroni/igae098.1799

**Published:** 2024-12-31

**Authors:** Leslie Hinyard, Stephanie Wladkowski, Verna Hendricks-Ferguson, Ruaa Al Juboori, Kathryn Coccia, Antonia Bennett, Cara Wallace

**Affiliations:** Saint Louis University, St. Louis, Missouri, United States; Bowling Green State University, College of Health & Human Services, Bowling Green, Ohio, United States; Saint Louis University, St. Louis, Missouri, United States; University of Mississippi, Oxford, Mississippi, United States; Saint Louis University, Chicago, Illinois, United States; University of North Carolina, Chapel Hill, North Carolina, United States; Saint Louis University, St. Louis, Missouri, United States

## Abstract

Live discharge from hospice has been referred to as the “forgotten care transition,” as it is understudied compared to other healthcare transitions. Following a hospice discharge, patients and families report feelings of grief and abandonment and 40% of patients die within 6 months of the discharge. However, no measures currently exist to help understand this care transition, an important gap for intervention and improvement. This study aimed to consider the utility of and relationship between the Care Transition Measure (CTM-15) and the Patient-Perceived Continuity of Care from Multiple Clinicians Measure among patients/caregivers following a discharge from hospice care. Though the CTM-15 measures the quality of preparation for post-hospital care, questions were modified to reference hospice instead of the hospital. Relational Continuity questions across 3 sub-scales—comprehensive knowledge of the patient, partnership & confidence, team relational continuity—were included from the Patient-Perceived Continuity of Care Measure, as relationship-based care is an important aspect of positive transitions during serious illness. 67 patients (or their primary caregiver) responded to questions following the experience of a live discharge from hospice. Patients were primarily female (75%) and white (78%), with just over half living at home (54%), and a mean age of 84. Study results found statistically significant, positive correlations between the CTM-15 and each of the subscales, indicating that as relational continuity increases, so does the positive perspective of transition quality. Participants also reported relevancy of the questions related to their live discharge experience, suggesting applicability of these tools among this population.

